# [Retracted] MicroRNA‑663 inhibits the proliferation and invasion of clear cell renal cell carcinoma cells by directly targeting PAK4

**DOI:** 10.3892/mmr.2023.12995

**Published:** 2023-04-12

**Authors:** Yingying Liu, Dan Jiao, Zhen Tian

Mol Med Rep 19: 711–718, 2019; DOI: 10.3892/mmr.2018.9652

Following the publication of this paper, it was drawn to the Editors’ attention by a concerned reader that certain of the data shown for the Transwell cell migration and invasion assays in Figs. 2C, 5D and 6D were strikingly similar to data appearing in different form in other articles by different authors, several of which have been retracted. Owing to the fact that the contentious data in the above article had already been published elsewhere, or were already under consideration for publication, prior to its submission to *Molecular Medicine Reports*, the Editor has decided that this paper should be retracted from the Journal. After having been in contact with the authors, they agreed with the decision to retract the paper. The Editor apologizes to the readership for any inconvenience caused.

